# Unstable Angina: Risk Stratification for Significant Coronary Artery Disease in The Era of High-Sensitivity Cardiac Troponin

**DOI:** 10.5334/gh.1286

**Published:** 2024-01-19

**Authors:** Luis Paiva, Maria João Vieira, Rui Baptista, Maria João Ferreira, Lino Gonçalves

**Affiliations:** 1 Faculdade de Medicina, Universidade de Coimbra, Portugal; 2 Serviço de Cardiologia, Centro Hospitalar e Universitário de Coimbra, Portugal; 3 Coimbra Institute for Clinical and Biomedical Research (iCBR), Universidade de Coimbra, Portugal; 4 Hospital Geral –Quinta dos Vales, 3041–801 Coimbra, Portugal; 5 Serviço de Cardiologia, Centro Hospitalar de Entre o Douro e Vouga, Santa Maria da Feira, Portugal; 6 Coimbra Institute for Biomedical Imaging and Translational Research, Universidade de Coimbra, Portugal

**Keywords:** unstable angina, non-ST-segment elevation acute coronary syndrome, high-sensitivity troponin, obstructive coronary artery disease, risk assessment

## Abstract

**Introduction:**

High-sensitivity troponin (hsTn) has a very high diagnostic accuracy for myocardial infarction (MI), and patients who were formerly diagnosed with unstable angina (UA) are being reclassified as having NSTEMI in the era of hsTn. This paradigm shift has changed the clinical features of UA, which remain poorly characterized, specifically the occurrence of obstructive coronary artery disease (CAD) and the need for myocardial revascularization. The main purpose of this study was to clinically characterize contemporary UA patients, assess predictors of obstructive CAD, and develop a risk model to predict significant CAD in this population.

**Methods:**

We conducted a retrospective cohort study of 742 patients admitted to the hospital with UA. All patients underwent coronary angiography. The endpoint of the study was the presence of obstructive CAD on angiography. The cohort was divided into two groups: patients with significant coronary artery disease (CAD^+^) and those without CAD (CAD^–^). We developed a score (UA CAD Risk) based on the multivariate model and compared it with the GRACE, ESC, and TIMI risk scores using ROC analysis.

**Results:**

Obstructive CAD was observed on angiography in 53% of the patients. Age, dyslipidemia, troponin level, male sex, ST-segment depression, and wall motion abnormalities on echocardiography were independent predictors of obstructive CAD. hsTn levels (undetectable vs. nonsignificant detection) had a negative predictive value of 81% to exclude obstructive CAD. We developed a prediction model with obstructive CAD as the outcome (AUC: 0.60).

**Conclusions:**

In a contemporary UA cohort, approximately 50% of the patients did not have obstructive CAD on angiography. Commonly available cardiac tests at hospital admission show limited discrimination power in identifying patients at risk of obstructive CAD. A revised diagnostic and etiology algorithm for patients with UA is warranted.

## Introduction

Non-ST-segment elevation acute coronary syndrome (NSTEACS) comprises two different clinical entities: non-ST-segment elevation myocardial infarction (NSTEMI) and unstable angina (UA). They differ primarily in whether ischemia is severe enough to cause myocardial damage and release significant amounts of cardiac biomarkers. Before the advent of sensitive troponin assays, UA hospital admissions exceeded those of ST-segment elevation myocardial infarction and accounted for 25%–50% of ACS cases [1].

Although the clinical setting has remained relatively unchanged over time, cardiac biomarkers have improved their performance, leading to changes in the diagnosis and management of UA [2]. High-sensitivity troponin (hsTn) has a very high diagnostic accuracy for myocardial infarction (MI), and patients who were formerly diagnosed with UA are being reclassified as NSTEMI in the era of hsTn. Although UA is a known predictor of cardiovascular adverse events [3,4], guidelines do not offer definite advice on how to stratify the risk for obstructive CAD or which patients should undergo coronary angiography to exclude unstable or severe CAD [5]. Literature regarding the risk stratification of NSTEACS is limited. Several studies have included patients with NSTEMI and UA [6,7,8], using biomarkers other than hsTn [6,7,8,9,10] (e.g., NTproBNP), echocardiogram [9], or coronary computed tomographic angiography findings [10] to assess prognosis. However, data specifically evaluating the occurrence of significant coronary artery disease (CAD) and the need for myocardial revascularization in UA patients are scarce.

The main purpose of this study was to evaluate obstructive CAD using coronary angiography, assess its predictors, and develop a risk model to predict significant CAD in a contemporary UA cohort.

## Materials and Methods

### Study design and settings

**Figure 1:**
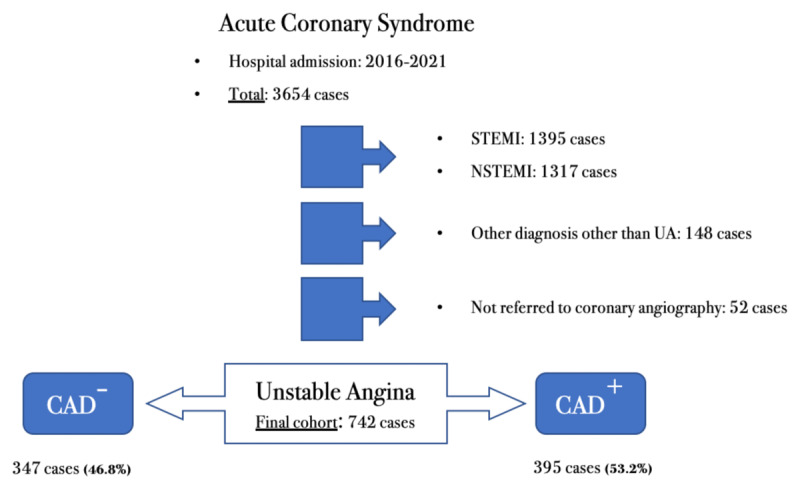
Flow diagram of the study. **CAD^–^**, no obstructive coronary artery disease; **CAD^+^**, presence of significant coronary artery disease; **UA**, unstable angina.

We conducted a retrospective, single-center, cohort study, including 3654 patients hospitalized for acute coronary syndrome, between January 1, 2016 and December 31, 2021. Patients were eligible for inclusion if they were more than 18 years old, and had a final clinical diagnosis of UA at hospital discharged, defined according to the universal definition of MI criteria (patients with chest discomfort in the absence of ST-segment elevation or left bundle branch block on the ECG and absence of hsTn elevation above the 99th percentile) [11]. The selected UA patients were reviewed for eligibility by two co-authors to exclude possible diagnoses other than UA. Furthermore, patients with UA who did not undergo coronary angiography for suspected obstructive CAD in the index event were excluded from the study. The final cohort included 742 patients with UA (Figure 1).

Baseline overall group characteristics with demographic, anthropometric, clinical, laboratory, echocardiographic, and angiographic data were obtained. All data were anonymized prior to statistical analyses. The study was conducted in accordance with the principles of the Declaration of Helsinki and approved by the local research ethics committee.

### Data collection

Blood samples were collected from all patients at admission for routine biochemical analysis. Troponin levels were measured using the Ortho-Clinical Diagnostics VITROS® Troponin I ES Assay (Rochester, NY, USA). The lower limit of sensitivity and detection of this test was 0.012 ng/mL and the 99th percentile was 0.034 ng/mL. An hsTn level of 0.033 ng/mL was considered as the upper limit for UA diagnosis in serial blood tests throughout the hospital stay. Patients were later divided into two categories according to maximum hsTn levels during the hospital stay: hsTn values ≤0.012 (undetectable) and hsTn values between 0.013 and 0.033 ng/mL (nonsignificant detection of troponin). Significantly elevated hsTn levels occurring after percutaneous coronary intervention (PCI; periprocedural MI) [12] were not considered an exclusion criterion in this study. Standard 12-lead ECGs were obtained at admission and during hospital stay. The presence of an ST-segment deviation, T-wave, Q-wave, conduction, or rhythm abnormalities was recorded. Routine transthoracic echocardiography (TTE) was performed during the index event (mean 1.9 ± 1.2 days after admission) using a Vivid 7 (GE Healthcare, Horton, Norway) ultrasound device.

Echocardiographic studies with standard views were performed as specified by the established guidelines [13]. The presence of segmental wall motion abnormality (WMA), defined as two or more adjacent segments with hypokinesia, akinesia, or dyskinesia, was recorded and the left ventricular ejection fraction (LVEF) was calculated using Simpson’s method. Coronary angiography during the hospital stay was considered a mandatory inclusion criterion, to make it possible to know the coronary epicardial disease. Those with significant CAD, defined as at least 70% diameter narrowing of a major coronary artery (or in the case of the left main coronary artery, an obstruction of at least 50% of its diameter), were considered for myocardial revascularization, either by percutaneous coronary intervention (PCI) or coronary artery bypass graft (CABG) [3]. The GRACE risk score was calculated according to the Fox model for death between hospital admission and 6 months [14]. The European Society of Cardiology (ESC) risk score for NSTE-ACS and Thrombolysis in Myocardial Infarction (TIMI) score risk for UA/NSTEMI were calculated as per guidelines [5,15].

### Study endpoint

The study endpoint was the presence of significant obstructive CAD on coronary angiography. The population was subsequently divided into patients with significant obstructive CAD (CAD^+^) and those without obstructive CAD (CAD^–^).

### Statistical analysis

Baseline characteristics were described as mean ± standard deviation for continuous variables and as counts and proportions for categorical variables. Continuous variables were compared using the *t*-test, and categorical variables were compared using Pearson’s chi-squared (χ^2^) test. We controlled for confounding effects by performing a multivariate logistic regression analysis to investigate the predictors of significant CAD. These data were used to estimate logistic regression models using the enter method to predict CAD^+^ patients. We developed a score (*UA CAD Risk*) based on a multivariate model of significant predictors of obstructive CAD in our cohort [16]. The *UA CAD Risk* discriminatory performance for obstructive CAD was compared with the GRACE risk score, the ESC, and TIMI guideline risk criteria by means of the area under the curve (AUC) on ROC analysis. Statistical analyses were performed using STATA (Stata Corp LP©, version 14.1). All reported differences had two-sided *p*-values <0.05.

## Results

The baseline clinical, echocardiographic, and analytical characteristics of patients with UA are shown in Table 1. The average age of the patients was 66 ± 11 years, and 69% (*n* = 506) were male. Regarding the 12-lead ECG, nearly 50% (*n* = 372) of patients had no relevant electrocardiographic ischemic changes and in those with abnormal ECG findings, ST-segment depression was found in 13% (*n* = 95) of patients. Coronary angiography performed during the hospital stay occurred mostly within the first day of hospitalization (mean 1.4 ± 1.5 days). Obstructive coronary artery disease (CAD^+^) was identified in 395 (53%) patients. Of these patients, 266 underwent PCI (67%), 18 underwent CABG (18%, *n* = 18), and 57 received medical treatment without any myocardial revascularization strategy (15%). The mean duration of hospital stay was 3.6 ± 4.5 days. No in-hospital death occurred in our cohort.

**Table 1:** Baseline characteristics of patients with clinical diagnosis of unstable angina.


	UNSTABLE ANGINA			

POPULATION	TOTAL n = 742	CAD^–^ n = 347 (47%)	CAD^+^ n = 395 (53%)	p

Male (%)	506 (69)	216 (62)	292 (74)	**<0.001**

Age (years ± SD)	66 ± 11.1	65 ± 11.5	67 ± 10.7	**0.003**

BMI (Kg/m^2^ ± SD)	28.1 ± 4.0	28.1 ± 4.2	28.1 ± 3.9	0.87

HTN (%)	622 (84)	279 (80)	343 (87)	**0.018**

Dyslipidaemia (%)	626 (84)	272 (78)	354 (90)	**<0.001**

Diabetes Mellitus (%)	260 (35)	104 (30)	156 (40)	**0.007**

Current/Past Smoking (%)	243 (33)	108 (31)	135 (34)	0.27

CKD (%)	75 (10)	30 (9)	45 (11)	0.22

Previous MI (%)	153 (21)	57 (16)	96 (24)	**0.008**

Previous PCI (%)	211 (29)	77 (22)	134 (34)	**<0.001**

Previous CABG (%)	39 (5)	0 (0)	39 (9.9)	**<0.001**

Aspirin (%)	569 (77)	251 (72)	318 (81)	**0.009**

P2Y_12_ receptor inhibitor (%)	266 (36)	4 (1)	262 (66)	**<0.001**

Statin (%)	621 (84)	293 (84)	328 (83)	0.61

ACE inhibitor or ARB (%)	622 (84)	279 (80)	343 (87)	**0.020**

Oral anticoagulant (%)	90 (12)	44 (13)	46 (12)	0.670

Beta-Blocker (%)	470 (63)	141 (41)	329 (83)	**<0.001**

Admission				

Killip-Kimball Class (%)				0.50

Class 1	700 (94.3)	330 (95.1)	370 (93.7)	

Class 2	41 (5.5)	17 (4.9)	24 (6.1)	

Class 3	1 (0.1)	0 (0)	1 (0.3)	

Class 4	0 (0)	0 (0)	0 (0)	

SBP (mmHg ± SD)	135 ± 21	133 ± 21	136 ± 22	**0.034**

Heart Rate (/min ± SD)	67 ± 13	69 ± 13	67 ± 12	0.19

Creatinine (µmol/L ± SD))	90.5 ± 65	84.8 ± 47	95.5 ± 78	**0.026**

NTproBNP (pg/ml ± SD))	637 ± 1875	685 ± 2389	580 ± 972	0.74

hsTn (ng/ml ± SD))	0.0157 ± 0.007	0.0145 ± 0.006	0.0168 ± 0.009	**<0.001**

ECG abnormalities (%)	372 (50)	167 (48)	205 (52)	0.32

ECG ST deviation (%)	95 (13)	33 (10)	62 (16)	**0.012**

LVEF (% ± SD)	55.5 ± 7.9	56.6 ± 7.2	54.4 ± 8.3	**<0.001**

Echocardiographic WMA (%)	223 (32)	73 (22)	150 (40)	**<0.001**

Grace Score				

In-hospital	111 (25.5)	107.7 (26.3)	112.2 (24.7)	**0.017**

6 months	94.9 (24.5)	92.1 (25.2)	97.3 (23.7)	**0.004**

Treatment				

PCI (%)	266 (36%)	0	266 (67%)	**<0.001**

CABG (%)	72 (10%)	0	72 (20%)	**<0.001**


**ACE inhibitors** – angiotensin-converting enzyme; **ARB** – angiotensin receptor blocker; **BMI** – body mass index; **CABG** – coronary artery bypass grafting; **CAD^+^** obstructive coronary artery disease; **CAD** – without obstructive coronary artery disease; **CKD** chronic kidney disease; **DBP** diastolic blood pressure; **ECG** 12-lead electrocardiogram; **HsTn** high sensitivity troponin; **HTN** arterial hypertension; **LVEF** left ventricular ejection fraction; **MI** myocardial infarction; **NTproBNP** N-terminal proB-type natriuretic peptide; **PCI** percutaneous coronary intervention; **SBP** systolic blood pressure; **WMA** wall motion abnormalities by transthoracic echocardiogram.

### Predictors of significant coronary artery disease

CAD^+^ patients were older (67 ± 11 vs. 64 ± 12 years, *p* = 0.03), predominantly male (74% vs. 62%, *p* < 0.001), and had a higher prevalence of medical comorbidities (Table 1), including dyslipidemia, hypertension, diabetes mellitus, and previous history of MI. At admission, they had higher systolic blood pressure, creatinine, and hsTn levels (0.017 ± 0.009 vs. 0.015 ± 0.006; *p* < 0.001) than those without significant coronary artery disease on angiography (CAD^–^). Patients with detectable hsTn levels (>0.012 ng/mL) had a higher risk of obstructive CAD (OR 1.79, 95% CI 1.3–2.5; *p* < 0.0001). The GRACE score was higher in patients with CAD^+^, namely in-hospital (112 ± 25 vs. 107 ± 26, *p* = 0.017) and 6-month (97 ± 24 vs. 92 ± 25, *p* = 0.004) risk scores. There were no significant differences between the two groups regarding the Killip–Kimbal class, heart rate, or NTproBNP levels at admission.

A normal 12-lead ECG did not exclude the presence of CAD in this population. However, CAD^+^ subjects showed a higher prevalence of ST-segment depression at admission (16% vs. 10%, *p* = 0.012) than CAD^–^ subjects, conferring a significantly higher risk of obstructive CAD (OR 1.8, 95% CI 1.1–2.8; *p* = 0.012). In the TTE at admission, CAD^+^ patients had a lower LVEF (54% ± 8% vs. 57% ± 7%, *p* < 0.001), more frequently presented with LVEF<50% (10% vs. 5%, *p* = 0.001), and LV WMA (21% vs. 10%, *p* < 0.001) than the CAD^–^ group. Patients presenting with LVEF ≥50% had a lower risk of obstructive CAD (OR 0.51, 95% CI 0.33–0.78; *p* < 0.002) and those with WMA had a higher risk of obstructive CAD (OR 2.4, 95% CI 1.7–3.3; *p* < 0.001).

**Figure 2:**
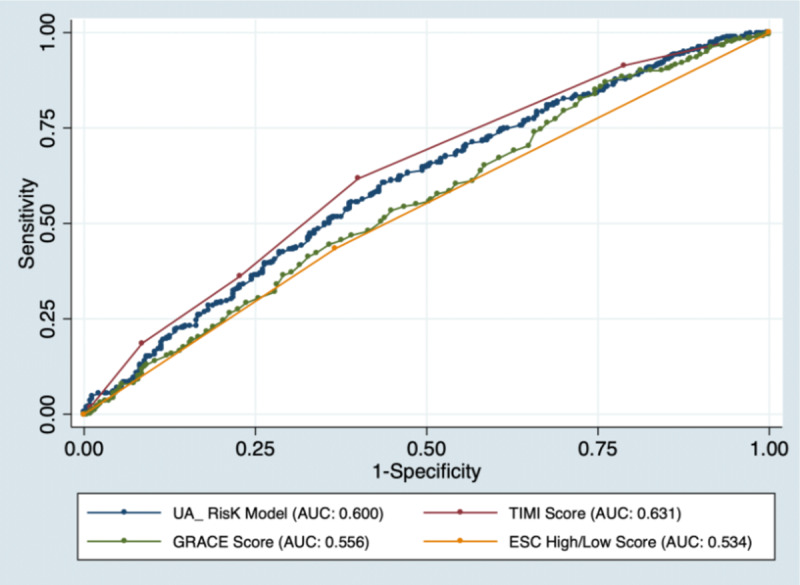
Receiver operating characteristic (ROC) curves with obstructive CAD as the outcome, comparing UA CAD Risk, GRACE, ESC, and TIMI risk scores. **ESC** European Society of Cardiology criteria, **GRACE** The Global Registry of Acute Coronary Events; **TIMI** Thrombolysis in Myocardial Infarction criteria; **UA** Unstable Angina. **CAD^–^**, no obstructive coronary artery disease; **CAD^+^**, presence of significant coronary artery disease; **UA**, unstable angina.

After adjusting for confounders, only sex, age, dyslipidemia, troponin level, ST-segment depression, and WMA were identified as incremental risk factors for predicting significant CAD on angiography (Table 2). We derived a prediction model using binary logistic regression, with obstructive CAD (CAD^+^) on angiography as the outcome. The variables included in the model were two demographic parameters (sex and age), one clinical (dyslipidemia) and one analytical variable (hsTn levels), and two cardiac diagnostic test findings (ST-segment depression, WMA), which are commonly assessed at hospital admission in UA subjects. Table 3 presents the weights of each factor in the model. The best-performing models were *UA CAD Risk* with an AUC of 0.60 (CI 95%, 0.56–0.64) and the TIMI with an AUC of 0.63 (CI 95%, 0.60–0.68). The GRACE (AUC: 0.55; CI 95%, 0.51–0.60) and ESC (AUC: 0.53; CI 95%, 0.49–0.56) risk scores for NSTE-ACS presented a lower performance for predicting obstructive CAD (Figure 2). Later, we derived a similar model using hsTn levels as a categorical variable (undetectable: ≤0.012 ng/dL vs. nonsignificant detection: 0.013–0.033 ng/dL), which presented a lower AUC of 0.59 (CI 95%, 0.55–0.63) by ROC analysis. Finally, we analyzed the accuracy of hsTn levels (undetectable vs. nonsignificant detection) in a univariate model, which presented a negative predictive value of 81.3% (CI 95%, 78.5%–84.9%) to exclude obstructive CAD in UA patients.

**Table 2:** Multivariate logistic regression for predictors of obstructive coronary artery disease in unstable angina patients.


	β COEF.	IC 95%	p

Male	0.532	0.186–0.879	**0.003**

Age	0.025	0.002–0.478	**0.032**

Arterial Hypertension	0.216	–0.225–0.657	0.337

Dyslipidaemias	0.648	0.190–1.107	**0.006**

Diabetes Mellitus	0.203	–0.134–0.540	0.239

Creatinine	0.001	–0.002–0.004	0.495

HsTn	31.935	9.956–53.923	**0.004**

WMA	0.308	0.097–0.519	**0.004**

ECG ST Deviation	0.745	0.191–1.298	**0.008**

In-hospital GRACE score	–0.006	–0.161–0.004	0.264


**ECG** 12-lead electrocardiogram; **hsTn** high sensitivity troponin; **MI** myocardial infarction; **WMA** wall motion abnormalities by transthoracic echocardiogram.

**Table 3:** The weight variables in the UA CAD Risk model.


MULTIVARIATE MODEL	β COEF. (95% CI)	p

Sex	0.519 (0.173–0.855)	0.003

Age	0.175 (0.003–0.032)	0.017

Dyslipidaemia	0.736 (0.290–1.182)	0.001

hsTn	32.399 (10.785–54.012)	0.003

WMA	0.331 (0.122–0.539)	0.002

ECG ST Deviation	0.579 (0.105–1.053)	0.017

Constant	– 2.745	


**ECG** 12-lead electrocardiogram; **hsTn** high sensitivity troponin; **MI** myocardial infarction; **WMA** wall motion abnormalities by transthoracic echocardiogram. **UA CAD Risk:** = e^(–0.122 + (0.175*Age) + (0.519*Sex) + (0.736*Dyslipidaemia) + (32.399*hsTN) + (0.331*WMA) + (0.579*ECG ST Deviation)) / 1 + e^(–0.122 + (0.175*age) + (0.519*Sex) + (0.736*Dyslipidaemia) + (32.399*hsTN) + (0.331*WMA) + (0.579*ECG ST Deviation)) Sex: male = 0; female = 1; Dyslipidaemia: absence = 0, presence = 1; WMA: absence = 0, presence = 1; ECG ST Deviation: absence = 0, presence = 1.

## Discussion

**Figure 3:**
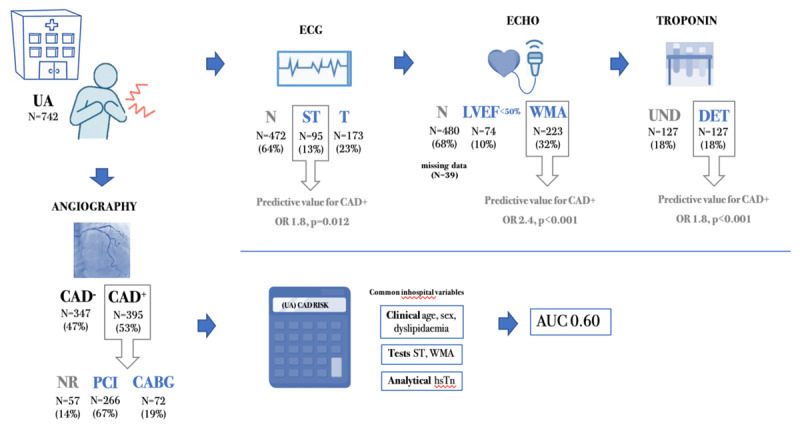
Main findings of the study. **CABG**, coronary artery bypass graft; **CAD^–^**, no obstructive coronary artery disease; **CAD^+^**, presence of obstructive coronary artery disease; **DET**, detectable hsTn levels; **ECG**, 12-lead electrocardiography; **ECHO**, transthoracic echocardiogram; **WMA**, ventricular wall motion abnormalities; **NR**, no myocardial revascularization; **N**, no relevant finding; **PCI**, percutaneous coronary intervention; **ST**, ST-segment changes; **T**, T-wave changes; **UA**, unstable angina; **UND**, undetectable hsTn levels.

We describe a cohort of contemporary UA patients who underwent coronary angiography to evaluate the presence of significant CAD. Our findings underline the limitations of commonly available tests at hospital admission (12-lead ECG, cardiac enzymes, and echocardiography) in stratifying the risk of obstructive CAD in UA patients. The *UA CAD Risk* and the TIMI score were the best-performing models, however, they presented limited discrimination power for identifying UA patients at risk of obstructive CAD (Figure 3).

UA is defined as myocardial ischemia at rest or with minimal exertion, in the absence of acute cardiomyocyte injury or necrosis [5]. It is a heterogeneous clinical entity with no specific symptoms or signs, ECG changes, or blood tests to support its diagnosis or identify patients that warrant hospital admission and myocardial revascularization. Patients with UA seem to present a lower risk of death and derive less benefit from intensified antiplatelet therapy and early invasive coronary strategies than patients with NSTEMI [17,18]. The systematic use of hsTn among patients presenting to the hospital with suspected ACS resulted in a relative 20% increase in the detection of MI and, therefore, a decrease in the diagnosis of UA [2]. Recent real-world data reported that 15%–32% of patients admitted with ACS had a final diagnosis of UA [19,20].

Guidelines do not provide a practical algorithm for managing UA after ruling out MI [3,5]. Considering risk stratification, it advises the conjugated use of clinical and ECG findings and hsTn levels to identify patients for early discharge and outpatient management or, in selected cases, indicate cardiac imaging tests or invasive coronary angiography. Overall, the diagnostic performance of chest pain characteristics for risk stratification is limited, and physical examination findings are often unremarkable in patients with UA [21]. Nonetheless in ‘stable angina’, typical anginal symptoms added to the risk scores are known to improve the prediction of obstructive CAD [22]. While 12-lead ECG at presentation is a useful tool for risk prediction, most of its prognostic power is derived from ST-segment deviation. The prognostic impact of isolated T-wave inversion is conflicting in the literature, and it does not alter the predictive value of associated ST-segment depression [23]. Although we found that nearly 50% of patients with a normal ECG presented with significant CAD, ST-segment depression was the only ECG finding associated with obstructive CAD in our cohort (twice the risk).

Cardiac biomarkers complement clinical assessment and 12-lead ECG in the diagnosis and risk stratification of ACS, as hsTn optimal thresholds for MI rule-out provided a negative predictive value of 99% in large validation cohorts. The finding of very low rates of MI at the index visit (<0.3%) or 30-day MACE (<0.5%) supports early discharge and outpatient management of UA cases, particularly in the absence of independent risk factors such as older age, previous MI, and eGFR < 60 mL/min/1.73 m^2^ [5]. The clinical risk in patients with UA appears to increase with increasing hsTn levels. UA patients with undetectable hsTn levels presented lower rates of death/MI at 1-year follow-up than those with detectable hsTn levels (<99th percentile) [24]. However, hsTn levels do not allow reliable prediction of obstructive CAD in UA setting. Accordingly, we found that 50% of patients with undetectable hsTn levels presented with obstructive CAD, though those with detectable troponin levels (<99th percentile) had approximately twice the risk of CAD on the coronary angiography. Moreover, undetectable hsTn levels showed considerable negative predictive value (81%) for significant CAD in our cohort.

TTE is recommended for patients presenting to the hospital with suspected ACS to evaluate abnormalities suggestive of myocardial ischemia/necrosis (regional/global LV function) and identify alternative conditions associated with chest pain. Additionally, in the absence of significant WMA, reduced LV regional function on strain imaging improves the diagnostic value of echocardiography [13]. Stress imaging can be considered in patients with a low-to-intermediate risk of ACS and is preferred over exercise ECG because of its superior diagnostic accuracy [5]. Nonetheless, stress imaging requires differentiated medical teams and is not continuously available to patients admitted to the emergency room. We found that routine TTE-derived data are useful for risk stratification of UA cases for obstructive CAD. In our study, most patients (85%) presented with an LVEF ≥ 50%, which seemed to halve the risk of significant CAD on angiography, and the presence of WMA increased the risk of obstructive CAD more than twice.

A UA diagnosis does not indicate the presence of obstructive CAD nor the need for a revascularization procedure. We found obstructive CAD in 53% of patients and a high rate of myocardial revascularization (PCI 67%, CABG 19%). Unfortunately, there are no specific recommended risk scores for evaluating the probability of obstructive CAD in ACS patients. Our prediction model (*UA CAD Risk*) using commonly available variables (age, sex, dyslipidemia, ST changes, WMA, and troponin values) and the GRACE, ESC, and AHA guideline risk criteria, which are the most recognizable prognostic risk scores for ACS, did not reliably predict significant CAD in our study [5,14,15]. Furthermore, hsTn levels (undetectable vs. nonsignificant detection) presented a poor discriminatory power to identify patients with obstructive CAD. Similarly, a previous study by Fladseth et al. [25] reported that 45% of UA patients presented with obstructive CAD, and GRACE score and ESC/ACC guideline risk criteria showed low diagnostic accuracy for CAD, with AUC of 0.59 and 0.58, respectively.

Previous clinical studies have reported models for risk stratification of patients with NSTE-ACS, some using one or more biomarkers [6,7,8], others combining biomarkers with echocardiogram or CCTA findings [9,10]. Tello-Montoliu et al. [6] included 358 patients admitted for NSTE-ACS and used troponin, NT-proBNP, C-reactive protein (CRP), and D-dimer levels to predict major adverse events. They reported that a multi-biomarker approach added prognostic value to the TIMI risk score. Previously, Omland et al. [7] described NT-proBNP as a powerful prognostic marker across the ACS spectrum. More recently, a study aimed to assess prognosis using a model with age, NT-proBNP, and ejection fraction (ABEF score) in patients with chronic coronary syndrome, who underwent PCI [9]. The authors found that a higher ABEF score was related to major cardiovascular events. Finally, Xia et al. [10] developed a risk model (BETTER) using several biomarkers (troponin, CRP, myeloperoxidase, ischemia-modified albumin, and NT-proBNP) and CCTA findings in patients with UA. They found that a combined approach (BETTER score) was the best strategy for risk stratification of patients with UA. However, these previous risk stratification models focused on prognosis and did not evaluate the occurrence of obstructive CAD or the need for myocardial revascularization in patients with UA. We sought to develop a clinical model (*UA CAD risk*) using variables that are routinely retrieved while assessing patients with UA (ST changes, WMA, and troponin values), which would not alter or add costs to the standard clinical practice in NSTE-ACS in the emergency room. Although bedside echocardiogram WMA evaluation is a more time-consuming and subjective assessment than a laboratory workup, there are no known biomarkers capable of properly recognizing significant CAD in patients with chest pain. Furthermore, echo-derived data have been reported to improve risk stratification in ACS [9]. Moreover, Xia et al. [10] described that coronary findings on CCTA (lesion, degree of stenosis, and epicardial fat) had a higher discriminatory power than biomarkers in predicting adverse events, suggesting that knowing the coronary anatomy of patients with UA may be a powerful prognostic marker.

Our results suggest that coronary angiography during hospitalization could have been avoided in nearly 50% of patients with a final diagnosis of UA, by improving the risk stratification and selection criteria. The prognostic implications of an invasive strategy in UA patients are unknown, and it is likely that UA patients will derive less benefit from a routine and early revascularization strategy than MI patients [5]. Previously, an individual patient-based meta-analysis in NSTE-ACS suggested that only patients with elevated biomarkers, GRACE score >140, age >75 years, and diabetes might benefit from an early invasive approach (<24 h) [20]. Moreover, guidelines recommend that patients with no recurrence of symptoms and no high-risk criteria (i.e., MI, dynamic ST/T segment changes, GRACE risk score > 140, resuscitated cardiac arrest, or cardiogenic shock) should be considered to be at low risk of short-term acute ischemic events and should follow the management algorithm of chronic coronary syndromes, preferably stress cardiac imaging over noninvasive anatomical testing [5,26]. Additionally, the differentiation of a true culprit lesion from bystander CAD in a UA setting can be challenging in some cases, and the benefits of revascularization procedures for chronic CAD are currently disputable [27].

Angina and nonobstructive CAD are associated with an increased risk of MACE [28] and may precede the development of epicardial lesions. Among patients with diabetes, those with nonobstructive epicardial disease but low coronary flow reserve (CFR) have a similar long-term prognosis to those with obstructive epicardial disease [29]. Discrepancies between coronary anatomy and symptoms or noninvasive tests often occur in clinical practice [30]. The limited diagnostic yield of coronary angiography may underestimate the functional significance of moderate stenosis or diffuse coronary narrowing, and microcirculatory function assessment using angiographic techniques is challenging. Since a systematic approach to explore microcirculatory or vasomotor coronary disorders is seldom implemented in catheterization laboratories, objective evidence of nonobstructive causes of ischemia is rarely established. The two main mechanisms of microvascular dysfunction are impaired microcirculation (measured by CFR and microcirculatory resistance index [IMR]) and arteriolar dysregulation (assessment of endothelial function with intracoronary acetylcholine). A recent randomized clinical trial (CorMicA) found that in patients with nonsignificant CAD, tailored treatment guided by CFR, IMR, and acetylcholine testing resulted in a significant reduction in anginal symptoms compared with conventional non-guided medical treatment [31]. β-Blockers, ACE inhibitors, and statins are used in patients with a dominant mechanism of microvascular dysfunction, whereas nitrates and calcium channel blockers are preferred in patients with vasospastic angina. A revised diagnostic algorithm for nonobstructive CAD with chest pain may be warranted, since a substantial number of UA patients will not present with obstructive epicardial coronary disease on angiography, as observed in our cohort.

## Future Perspectives

Risk stratification of obstructive CAD in the UA setting using traditional clinical, electrocardiographic, and echocardiographic parameters is not expected to improve in the future. Diagnostic strategies using stress imaging tests (e.g., stress echocardiography) may significantly add to the decision to refer patients to the catheterization laboratory. However, stress imaging is only considered for patients without ischemic changes on a 12-lead ECG and free from chest pain for several hours. Moreover, the widespread implementation of stress tests in UA would add complexity to chest pain rapid algorithms and more constraints in the emergency room. The best algorithm for UA management is unknown and warrants further clinical studies. The etiological characterization of UA cases (epicardial disease, microvascular dysfunction, and structural disease) may allow for more efficient treatment of anginal symptoms. Future studies should also address whether UA patients with no high-risk findings can be safely discharged without further cardiac testing, and continue their clinical investigation on an outpatient basis.

## Study Limitations

Our retrospective study was limited by a potential registration bias and the influence of putative unmeasured confounders. Regarding medications at hospital admission, the available data did not allow to ascertain patient compliance prior to the index event or to determine the duration of medication use. To minimize observer bias, the data collection was blinded to the angiographic results. As there are no defining clinical characteristics of UA, its diagnosis is supported by chest pain and the absence of acute cardiac injury markers (12-lead ECG and hsTn). The heterogeneity of UA clinical presentation makes it a subjective clinical entity and prone to interobserver bias. The definite exclusion of chronic coronary syndromes would only be possible with a clear demonstration of a culprit coronary artery plaque, namely through intracoronary imaging. Moreover, our study consisted of a very selected cohort of patients who underwent coronary angiography to enable a standard CAD assessment. Although real-life studies have several limitations, they have the advantage of representing this challenging population in daily clinical practice.

## Conclusions

In a contemporary UA cohort, approximately 50% of the patients did not have obstructive CAD on angiography. Commonly available cardiac tests at hospital admission show limited discrimination power in identifying patients at risk of obstructive CAD. A revised diagnostic and etiology algorithm for patients with UA is warranted.

## Data Accessibility Statement

The data underlying this article are available in the article.
